# The importance of local epidemic conditions in monitoring progress towards HIV epidemic control in Kenya: a modelling study

**DOI:** 10.1002/jia2.25203

**Published:** 2018-11-28

**Authors:** Sarah‐Jane Anderson, Geoffrey P Garnett, Joanne Enstone, Timothy B Hallett

**Affiliations:** ^1^ Department of Infectious Disease Epidemiology Imperial College London London UK; ^2^ The Bill and Melinda Gates Foundation Seattle WA USA; ^3^ Division of Public Health and Epidemiology The University of Nottingham Nottingham UK

**Keywords:** programme evaluation, population surveillance, models, theoretical, HIV Infections, epidemiology, forecasting

## Abstract

**Introduction:**

Setting and monitoring progress towards targets for HIV control is critical in ensuring responsive programmes. Here, we explore how to apply targets for reduction in HIV incidence to local settings and which indicators give the strongest signal of a change in incidence in the population and are therefore most important to monitor.

**Methods:**

We use location‐specific HIV transmission models, tailored to the epidemics in the counties and major cities in Kenya, to project a wide range of plausible future epidemic trajectories through varying behaviours, treatment coverage and prevention interventions. We look at the change in incidence across modelled scenarios in each location between 2015 and 2030 to inform local target setting. We also simulate the measurement of a library of potential indicators and assess which are most strongly associated with a change in incidence.

**Results:**

Considerable variation was observed in the trajectory of the local epidemics under the plausible scenarios defined (only 10 of 48 locations saw a median reduction in incidence of greater than or equal to an 80% target by 2030). Indicators that provide strong signals in certain epidemic types may not perform consistently well in settings with different epidemiological features. Predicting changes in incidence is more challenging in advanced generalized epidemics compared to concentrated epidemics where changes in high‐risk sub‐populations track more closely to the population as a whole. Many indicators demonstrate only limited association with incidence (such as “condom use” or “pre‐exposure prophylaxis coverage”). This is because many other factors (low effectiveness, impact of other interventions, countervailing changes in risk behaviours, etc.) can confound the relationship between interventions and their ultimate long‐term impact, especially for an intervention with low expected coverage. The population prevalence of viral suppression shows the most consistent associations with long‐term changes in incidence even in the largest generalized epidemics.

**Conclusions:**

Target setting should be appropriate for the local epidemic and what can feasibly be achieved. There is no one universally reliable indicator to predict future HIV incidence across settings. Thus, the signature of epidemic control must contain indications of success across a wide range of interventions and outcomes.

## 1 Introduction

From early in the HIV pandemic, efforts have been made to measure and disseminate indicators describing the state of the pandemic and the risks of further spread of the virus. More recently, targets or goals have been promoted to galvanize support and to steer efforts to reduce AIDS deaths and HIV infections. Indicators have then been used to track progress in meeting these goals 1, 2. Most recently, Joint United Nations Programme on HIV/AIDS (UNAIDS) has established “Fast Track” commitments for 2020 of reducing the yearly number of new infections and AIDS deaths globally each to 500,000, on course to reduce further to 200,000 per year by 2030, which has been called “ending the epidemic” 3. This is equivalent to a 90% reduction in incidence from 2010 levels by 2030. These overarching goals, adopted by the UN General Assembly in 2016, are supposed to be achieved by reaching a set of intervention coverage targets. The relationship between the goals for infections and deaths and necessary interventions to reach them were established using the GOALS model of the spread of HIV infection 4. These Fast Track targets for new infections and deaths were based on the aggregate of simulations of national HIV epidemics where the intervention achievements, both for treatment and prevention, were assumed to reach 90% coverage in most cases.

In parallel to this process of establishing targets, WHO led an extensive consultation process to generate Strategic Information Guidelines, which highlighted the importance of specific “indicators.” These indicators are a mix of epidemiologic, programmatic and social measures of the status of the HIV epidemic and the efforts made to halt its spread 5. The function of indicators is to assess whether we are on track to control the epidemic, and if not, which aspects of the response require attention. The resulting “10 global indicators” in WHO's strategic guidelines include a number of interrelated epidemiological measures: HIV prevalence, HIV deaths and HIV incidence, and then focus on treatment with the coverage of diagnosis, linkage, treatment, retention in treatment and viral suppression; in addition, there is the input of domestic financial resources and “prevention” coverage 5. Building on these guidelines and the UNAIDS Fast Track Targets, new guidelines on Global AIDS Monitoring specified 68 indicators to track the HIV epidemic 6.

What has been lacking in recent policies and guidelines is a detailed exploration of the relationship in different settings between the indicators to be measured and the impact on HIV incidence, and of the interaction and relative importance of different variables.

It is possible in theory to understand how different variables that could be measured will impact HIV incidence. By definition, an infectious disease is spread via infectious contacts, so the determinants of these contacts (how long people are infectious for, who they have effective contacts with and the likelihood of transmission taking place) must determine spread of infection and should therefore be appropriate indicators. Thus, many early studies of HIV spread explored numbers of sexual partners, patterns of sexual partner choice and frequency of unprotected sex with partners 7, the presence of other sexually transmitted diseases 8, 9, and more recently, measures of viral load 10, which capture the contact pattern and the transmission probability of infection. However, the wide diversity of epidemic characteristics 11, 12, 13, the interaction between multiple groups with different risks and the range of responses to the epidemic complicate the application of simple theoretical insights to understand the range of indicators available to track HIV epidemics. To explore how epidemiological context and the interaction of variables influences the relationship between indicators and HIV incidence, we have analysed a model describing the HIV epidemic in each of the counties of Kenya. Our aim was to identify appropriate targets for the change in incidence across settings and assess how well a range of variables indicate the expected incidence of HIV in each location.

## 2 Methods

Ethical approval was not required for this modelling study. All model inputs were taken from published publicly available data sources.

This analysis seeks to explore how the indicators proposed for monitoring HIV epidemic control are likely to perform considering (1) the local diversity and (2) the complexity of the HIV epidemic and response including the uncertainty in the potential effect sizes of different interventions. We use location‐specific mathematical models, representing the diverse counties of Kenya, to project the future of the epidemic in each location, and through simulating the measurement of key indicators from the model can mimic surveillance of the epidemic. Kenya provides a good example country to explore well‐documented distinct epidemics that are driven by different population groups across the counties.

The models were calibrated to represent 48 Kenyan locations, the different counties and major cities, at baseline, and adapted from models described elsewhere 14. These models simulate heterosexual and homosexual transmission of HIV in a risk stratified population, across which it is possible to apply a variety of HIV prevention interventions and modulate the features of the treatment programme. A number of extensions to our preexisting model 14 were made to allow for a wider range of possible future outcomes and indicators to monitor (described in the Supporting Information). The modelled population in each location include men who have sex with men (MSM) who may engage in both heterosexual and homosexual partnerships, female sex workers (FSWs) and the general population of heterosexual men and women. In each location, the importance of these groups was established by removing them from simulations and comparing the epidemic with and without them. This provides the complete proportion of infections attributable to the group.

For each location, we simulated a range of plausible future epidemics for HIV incidence. Our goal was to vary as widely as credible the input variables, including behaviours and the coverage of interventions to see how they influenced incidence. We also simulated the measurement of a large library of potential indicators and see in statistical analysis how well they track the epidemic. We use geographically specific mathematical models so we can explore how local heterogeneity influences target setting and indicator strength (i.e. ability to predict change incidence over time). In this way, they allow for exploration of how best to monitor the epidemic dependent on the local characteristics.

To reflect uncertainty in the future course of the epidemic and the success of the response, we define many different plausible future scenarios for each modelled location (“the universe of plausible projections”). Looking across these plausible scenarios, we can assess how the magnitude of reduction in incidence varies across locations and which indicators are likely to provide the best guide to these changes. Plausible changes in the underlying sexual behaviours, prevention programmes and the characteristics of the treatment programme were described through defining bounds for input parameter values governing these features (Table 1). Parameters describing behaviour change may reflect an increase in risk behaviours in the population or modest reductions in risk behaviour assumed to be reflective of a behaviour change intervention. Latin Hypercube Sampling was used to draw 10,000 parameter combinations uniformly between these bounds, to describe changes in the epidemic and response from the year 2015 to generate the “universe of plausible projections.” For each modelled location, the change in incidence from the year 2015 to 2030 was estimated for each projection of the “universe of plausible projections.” This allows us to compare how the local epidemics respond to the set of plausible scenarios we define. A panel of different indicators were “output” from the model (listed in Table 2). A linear regression model was used to assess the relationship between the changes in each indicator individually in the short term (2015 until 2020) and the change in incidence in the long term (2015 until 2030). The adjusted R^2^ value gives the goodness of fit of the resulting model to the data (where a value of 1 suggests that all variation in modelled incidence trajectory is explained by the indicator).

**Table 1:** Minimum and maximum values of model parameters which are varied to give the “Universe of Plausible Projections”

	Minimum	Maximum
Behavioural parameters		
Proportion change in partner change rate in FSW (from “baseline” in 2015)	−0.5 (50% reduction in partner change rate)	0.1 (10% increase in partner change rate)
Proportion change in partner change rate in low‐risk women (not FSW) (from “baseline” in 2015)	−0.2 (20% reduction in partner change rate)	0.1 (10% increase in partner change rate)
Proportion change in partner change rate in heterosexual men (from “baseline” in 2015)	−0.2 (20% reduction in partner change rate)	0.1 (10% increase in partner change rate)
Proportion change in partner change rate in MSM (from “baseline” in 2015)	−0.5 (50% reduction in partner change rate)	0.1 (10% increase in partner change rate)
Proportion change in condom use (casual partnerships) (from “baseline” in 2015)	−0.2 (20% reduction)	0.1 (10% increase)
Proportion change in condom use (low‐risk partnerships) (from “baseline” in 2015)	−0.2 (20% reduction)	0.1 (10% increase)
Proportion change in condom use (commercial partnerships) (from “baseline” in 2015)	−0.5 (50% reduction)	0.1 (10% increase)
Proportion change in condom use (partnerships in MSM) (from “baseline” in 2015)	−0.5 (50% reduction)	0.1 (10% increase)
Programmatic parameters		
Circumcision rate (an additional intervention‐ capped so that only “on” if circumcision in the population is at <80%)	0	0.2 (per year)
PrEP coverage in FSW	0	0.5 (50% coverage)
PrEP coverage in low‐risk women	0	0.25 (25% coverage)
PrEP coverage in heterosexual men	0	0.25 (25% coverage)
PrEP coverage in MSM	0	0.5 (50% coverage)
Reduction in efficacy due to incomplete PrEP adherence	0.6	0.9
Treatment programme parameters		
Proportion of those entering ART from low CD4 (<200) with good adherence	0.6	0.95
Proportion of those ART from high CD4 (>200) with good adherence entering	0.3	0.9
Rate of drop out from HIV programme	0.001 (per year)	0.04 (per year)
Proportion of those who drop out that can reinitiate	0.4	0.7
Net survival time on ART	17 years	40 years
Proportion who can drop out from the treatment programme	0.5	1
Proportion of FSW who receive early ART (all CD4)	0.4	0.8
Proportion of low‐risk women (not FSW) who receive early ART (all CD4)	0.2	0.4
Proportion of heterosexual men who receive early ART (all CD4)	0.2	0.4
Proportion of MSM who receive early ART (all CD4)	0.4	0.8

ART, antiretroviral therapy; FSW, female sex workers; MSM, men who have sex with men; PrEP, pre‐exposure prophylaxis.

**Table 2:** List of indicators simulated in the model^a^

	WHO global indicator?
1. ART programme	
Change in HIV prevalence (not virally suppressed)	
Change in HIV prevalence (not virally suppressed) in MSM	
Change in HIV prevalence (not virally suppressed) in men	
Change in HIV prevalence (not virally suppressed) in women	
Change in HIV prevalence (not virally suppressed) in FSW	
Change in proportion of the population on ART	WHO 6: percentage on ART
Change in number on ART	
Change in number on ART (MSM)	
Change in number on ART (men)	
Change in number on ART (women)	
Change in number on ART (FSW)	
Change in proportion who are eligible who are on ART (eligibility defined here based on “treatment policy in the model”)	
Change in proportion of those HIV positive who are on ART	
Change in reported ART coverage (CD4 < 200)	
Change in number eligible who are not on ART	
Change in the fraction of those initiating with CD4 category (0 to 200)	
Change in the fraction of those initiating with CD4 category (200 to 350)	
Change in the fraction of those initiating with CD4 category (350 to 500)	
Change in the fraction of those initiating with CD4 category (500+)	
Change in proportion retained and surviving after 12 months on ART	WHO 7: percentage retained and surviving on ART 12 months after initiation
Change in percentage of those on treatment who are virally suppressed (<12 months)	WHO 8: percentage on ART who are virally suppressed
Change in percentage of those on treatment who are virally suppressed (>12 months)	
2. Changes in behaviour	
Proportionate change in partner change rate in FSW	
Proportionate change in partner change rate in low‐risk women	
Proportionate change in partner change rate in heterosexual men	
Proportionate change in partner change rate in MSM	
Overall change in the partner change rate in the population	
Change in condom use at last sex (casual partnerships)	
Change in condom use at last sex (low‐risk partnerships)	
Change in condom use at last sex (commercial partnerships)	WHO 3: percentage condom use in key populations
Change in condom use at last sex (partnerships in MSM)	WHO 3: percentage condom use in key populations
3. Programmatic indicators	
Change in circumcision coverage	
Change in percentage using PrEP in priority populations	
Change in PrEP coverage in FSW	
Change in PrEP coverage in low‐risk women	
Change in PrEP coverage in heterosexual men	
Change in PrEP coverage in MSM	
Change in PrEP efficacy (i.e. due to changes in adherence)	

ART, antiretroviral therapy; FSW, female sex workers; MSM, men who have sex with men; PrEP, pre‐exposure prophylaxis.

a Those indicators which are included in the WHO global indicator list are indicated.

## 3 Results

**Figure 1:**
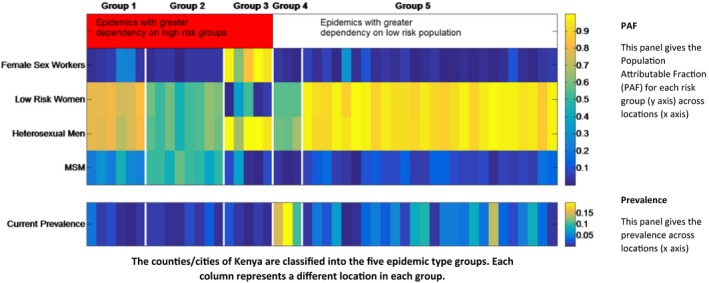
Classification of the forty‐eight modelled locations into five epidemic types This figure describes the classification of counties according to their epidemiological characteristics for each location (horizontal axis) of the five epidemic types (groups 1 to 5 delineated with vertical white lines). The top panel shows the PAF by sub‐population (vertical axis) across locations (horizontal axis) in each epidemic group and the bottom panel shows the HIV prevalence across locations (horizontal axis) in each epidemic group. Here, groups 1 to 3 represent those epidemics with a relatively high dependency on transmission from high‐risk groups. Group 1 represents those epidemics with high PAF values in both general and high‐risk populations. Group 2 represents those epidemics with large PAF values for MSM and group 3 represents FSW‐driven epidemics. Groups 4 and 5 describe those epidemics with lower contributions from higher risk groups and a greater dependency on the general population. Group 4 represents more established epidemics and very high prevalence settings. Group 5 represents those epidemics with most transmission in the general population but with generally lower population prevalence than in group 4. FSW, female sex workers; MSM, men who have sex with men; PAF, population attributable fraction.

Locations are grouped into different epidemic types according to the patterns of HIV transmission in the population and intensity of the epidemic, which we use when reporting results of our analysis (Figure 1). The Population Attributable Fraction (PAF) for each sub‐population in each location was calculated through removing transmission from the sub‐population of interest and examining the difference in the number of new infections occurring in the total population 15. Epidemics were classified into groups using k‐means clustering based on the PAF values across sub‐populations and HIV prevalence in each location. The characteristics of the groups are presented in Figure 1 and Table S1; we can see that locations within each group are relatively homogeneous in their characteristics. Groups 1 to 3 represent those epidemics with a relatively high dependency on transmission from high‐risk groups. Groups 4 and 5 describe those epidemics with lower contributions from higher risk groups and a greater dependency on the general population. Group 4 represents more established epidemics and very high prevalence settings. Group 5 represents those epidemics with most transmission in the general population but with generally lower population prevalence than in group 4.

### 3.1 How do global goals for a reduction in incidence translate to local epidemic contexts?

**Figure 2:**
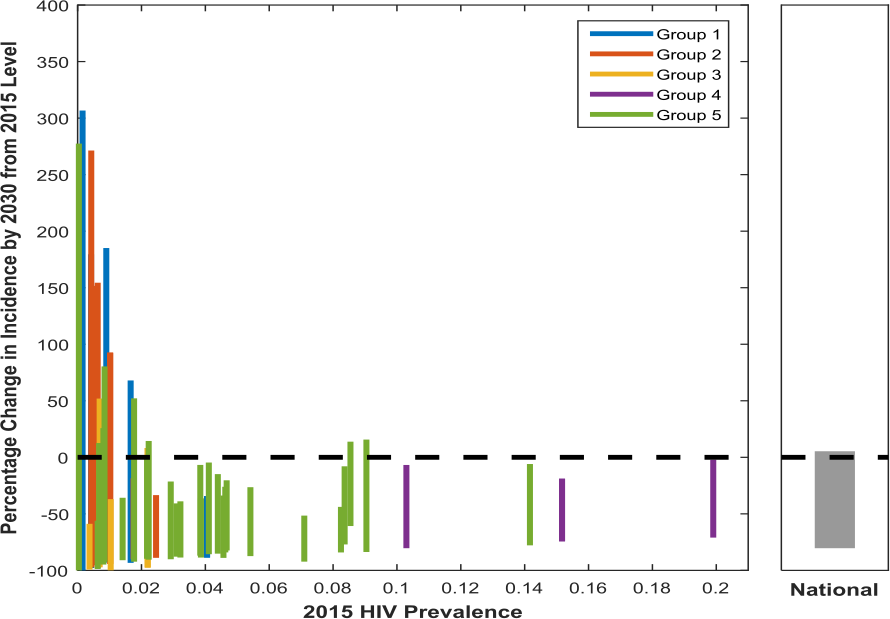
Change in incidence observed by 2030 from 2015 levels for each modelled location (left panel) and nationally (Right Panel) Each line corresponds to a different location, with the maximum and minimum value corresponding to the maximum and minimum change in incidence between 2015 and 2030 across the modelled future scenarios (the universe of plausible projections). The colour of each plotted location corresponds to the epidemic group it belongs to. Here, groups 1 to 3 represent those epidemics with a relatively high dependency on transmission from high‐risk groups. Group 1 represents those epidemics with high PAF values in both general and high‐risk populations. Group 2 represents those epidemics with large PAF values for MSM and group 3 represents FSW‐driven epidemics. Groups 4 and 5 describe those epidemics with lower contributions from higher risk groups and a greater dependency on the general population. Group 4 represents more established epidemics and very high prevalence settings. Group 5 represents those epidemics with most transmission in the general population but with generally lower population prevalence than in group 4. FSW, female sex workers; MSM, men who have sex with men; PAF, Population Attributable Fraction.

Figure 2 presents the percentage change in incidence observed by 2030 relative to 2015 levels (*y*‐axis) for each modelled location plotted against 2015 HIV prevalence (*x*‐axis). Each line gives the range (i.e. the minimum and maximum) in change in incidence we see for each location across all the plausible modelled scenarios (the “universe of plausible projections”). The different colours of each plotted location correspond to the epidemic group it belongs to (defined previously in Figure 1).

The national result (right‐hand panel) demonstrates how examining national level changes alone will mask the considerable heterogeneity observed across subnational locations (left panel, with each bar representing a different modelled location). The size of the projected change in incidence is extremely variable between modelled locations highlighting the need for local targets to reflect this heterogeneity. The median change in incidence across locations varies from a 26% reduction in incidence to a 100% reduction, and only 10 locations have a median reduction of >80%.

Those epidemics more concentrated in high‐risk groups (groups 1 to 3) have lower initial population HIV prevalence (left‐hand side of plot). These locations generally see a much greater range in percentage change in incidence across the modelled future scenarios between 2015 and 2030 (however, this relative change corresponds to a comparatively smaller absolute change due to lower initial incidence in these locations). We see some increases in incidence projected (positive percentage change) caused by some input variables representing increases in risk behaviours. This wider range in change in incidence observed is despite the fact that we would expect “higher risk groups” such as MSM and FSWs to have a higher R0 (basic reproduction number: expected number of secondary cases produced by an infection in a wholly susceptible population). But the higher coverage of interventions assumed in these groups combined with the less complex patterns of transmission in the total population means that programmes can be more impactful. In contrast, group 4 locations, which are the highest prevalence generalized settings, show more modest declines in incidence and less variation across the universe of plausible projections. This is because it is more difficult to alter the trajectory of established generalized epidemics with complex patterns of transmission.

### 3.2 Which indicators are predictive of changes in incidence?

**Figure 3:**
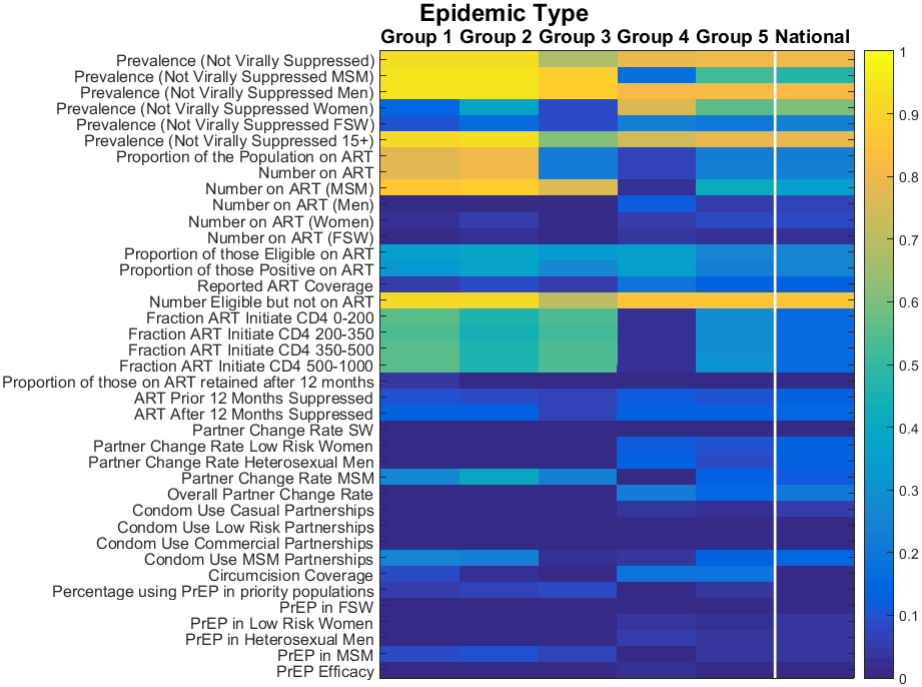
Strength of each indicator in predicting long‐term changes in incidence for each epidemic type Dark colours are those indicators with low adjusted R^2^ values (blue), light colours are those indicators with high adjusted R^2^ values and that perform much more strongly (yellow). Group 1 represents those epidemics with high PAF values in both general and high‐risk populations. Group 2 represents those epidemics with large PAF values for MSM and group 3 represents FSW‐driven epidemics. Groups 4 and 5 describe those epidemics with lower contributions from higher risk groups and a greater dependency on the general population. Group 4 represents more established epidemics and very high prevalence settings. Group 5 represents those epidemics with most transmission in the general population but with generally lower population prevalence than in group 4. FSW, female sex workers; PAF, Population Attributable Fraction; MSM, men who have sex with men.

We examined the performance of a panel of different indicators in how well they reflect changes in incidence over time (2015 to 2030) and thus are predictive of long‐term control of the epidemic. Figure 3 gives the results for each of the indicators across locations of the five epidemic groups. The colour of the panel indicates the median adjusted R^2^ value across locations of each category that is the goodness of fit of the resulting model to the data (where a value of 1 suggests that all variation in modelled incidence trajectory is explained by the indicator).

Looking across the entire figure, we can see that while certain indicators are well associated with changes in incidence (yellow), there are many which provide only a weak association (blue). Indicators which describe specific components of an intervention programme (such as “Condom use” or “PrEP coverage”) tend to have only a low association with incidence. This is because many other factors (low effectiveness, the impact of other interventions, countervailing changes in risk behaviours, etc.) can confound the relationship between interventions and their ultimate long‐term impact. This is especially true where the magnitude of impact from an intervention is small due to low range of expected coverages. Thus, the signature of epidemic control must contain indications of success across a wide range of interventions and outcomes.

In contrast, indicators that best predict declines in incidence are those related to the number of persons living with HIV who are likely to transmit (i.e. are not virally suppressed) (Figure 3). Such metrics combine a measure of burden, and so capture the net effect of interventions already in process, and a feature closer to the proximate determinants of transmission that is the number of infected people, and so are less readily confounded by other factors. In particular, the prevalence of non‐viral suppression, particularly for men, shows the most consistent association with changes in incidence across locations. This is because it is closer still to the actual determination of transmission.

As expected, there are also substantial differences in the strength of association with incidence across the different types of epidemic. In particular, indicators relating to the MSM population have a very strong association with incidence in those epidemic types with a high dependency on MSM (groups 1 and 2; R^2 ^> 0.9). However, these indicators are not consistently strong across all epidemic types (groups 3, 4 and 5), and in FSW‐driven and generalized epidemics show only limited association with incidence. In contrast, in the FSW‐driven epidemics (group 3), fewer indicators show strong signals, and the pattern of indicators of higher strength shows features of both the MSM‐driven (groups 1 and 2) and generalized epidemics (groups 3 and 4). Meanwhile, indicators pertaining to key populations have weaker performance in generalized epidemics (groups 4 and 5).

Predicting future changes in incidence is more challenging overall in FSW‐driven (group 3) and generalized epidemics (groups 4 and 5) because the largest declines require many conditions to be favourable (high coverage and effectiveness of all interventions), which no single indicator can capture adequately. Less complex patterns of transmission of infection (i.e. concentrated in a smaller fraction of the population) as seen in the MSM‐driven epidemics makes prediction of changes in incidence less challenging.

## 4 Discussion

The ambitious calls to “Fast Track” the end of the epidemic will need to be accompanied by appropriate targets and stringent monitoring to track and expedite progress towards epidemic control. This analysis has provided a number of important insights into defining local targets and choosing indicators to monitor changes in incidence in the population. Firstly, as the future epidemic trajectory varies considerably between modelled locations in Kenya under the plausible scenarios presented, target setting should adapt to what is achievable in each local setting. Secondly, the ability of each indicator to predict changes in incidence over time is also heterogeneous across modelled locations. Few indicators are consistently strong predictors of epidemic control across all settings. This is because the different epidemic types will influence which set of indicators perform the strongest. Finally, many of the indicators demonstrate only limited association with changes in incidence. This is due to many potentially countervailing factors which can alter the epidemiology and thus the future impact achievable through interventions. This highlights the requirement to maintain a sustained and multifaceted approach in designing programmes to meet the complexity of the epidemic and achieve reductions in incidence. Measures of the population prevalence of viral suppression show more consistent associations with long‐term changes in incidence across locations.

The variability in the trajectories of the local epidemic under the universe of plausible scenarios defined here has important implications for future target setting. Despite the same scenarios of future change being applied in all locations, only 10 of 48 locations would reach a target of 80% reduction in incidence. Our results suggest that careful consideration of how global goals for a reduction in incidence translate to local epidemic contexts is critical and that a single target may not be achievable or appropriate to all locations. Factors such as a higher initial burden, complex patterns of transmission in the population and the existing coverage of interventions will influence the impact of HIV prevention programmes in a local context.

Furthermore, how progress is measured and what indicators should be closely monitored will also depend on the local setting. Key populations are critical in concentrated epidemics, and specific guidelines are available for monitoring these groups 16, 17. In generalized epidemics, key populations also play an important role in transmission 18, but a more comprehensive picture of the entire population is needed. Indicators reliably associated with future changes in incidence are fewer in advanced generalized epidemics (groups 4 and 5).

Across all settings, it is likely that a battery of indicators may be required for a complete picture of the epidemic as many of the indicators considered here gave only a weak indication of future changes in incidence alone. Of all the indicators considered, the prevalence of viral suppression performs best in predicting future changes in the epidemic. This supports previous proposals to use “community viral load,” when defined this way as an important measure of potential transmission in the population as a whole (compared to looking only at the clinic population) 19, 20. Furthermore, such an indicator allows us to condense and capture the overall success of the 90‐90‐90 cascade treatment target.

The variation in impact across subnational regions also means that national trends of any indicator are likely to mask specific and important local trends, as is the case of prevalence data from antenatal clinics in Zambia 21. This highlights the need for greater availability of disaggregated indicators across subnational regions to understand detailed changes in the epidemic.

A number of further extensions to these analyses could be considered. Importantly, we do not explore the indicators disaggregated by age groups, which may give a stronger measure of new infections in the population. Alternative model structures, with different representations of risk in the population or different modes of action of interventions may provide different insights. The model results presented here are based on assuming that the coverage and effectiveness of interventions is held constant over time; indicators may not perform as well if this was not the case. We did not represent migration between the modelled locations in this study, yet resultant shifts in the demographic, behavioural and epidemiological characteristics or changes in the coverage of services, may alter the course of the epidemic and the set of indicators most predictive of future incidence. The epidemiological analysis presented here focuses on HIV prevention targets, and evaluates indicators for their ability to predict reductions in HIV incidence in the population. Future analyses could explore the utility of indicators in evaluating progress towards other public health targets, including the maximization of measures of population health (quality‐adjusted life years) or reduction in burden of disease (disability‐adjusted life years) which could capture HIV prevention, treatment and health system progress.

It must also be highlighted that this modelling study cannot account for other factors which will influence local target setting and the choice of indicators in reality. Local target will involve numerous considerations in addition to the epidemic characteristics, such as feasibility of providing services and funding available. The source and quality of the data used to measure each indicator must be considered, whether they are modelled or directly measured, and whether observed data is robust in terms of geographical resolution, representativeness, the frequency of measurement, changes in surveillance, any reporting biases and the degree of completeness. Indicators will in practice be most useful if, in addition to reflecting changes in incidence, they are measurable and actionable in HIV programmes. The method of testing, or the populations included may change over time, complicating interpretation of observed trends 22. Frameworks to evaluate the population health benefits of measuring different indicators to inform decisions have been explored elsewhere 23, including in the design of HIV prevention programmes in Zambia 24.

## 5 Conclusions

Because of the considerable heterogeneity in the epidemic at local levels, and the different strategic approach and intensity of the response, both target setting and indicator choice should be performed at local level. Many of the widely used indicators based on programme parameters may not independently provide a reliable guide to the extent of incidence decline that will be seen, but combinations of indicators that combine measures of burden and proximate determinants of transmission (i.e. non‐suppression of viral load) may be among the more useful of the proposed indicators. Our results suggest that there is no one target for control, or one indicator to measures progress, which is appropriate to all epidemic settings.

## Competing Interest

SJA reports personal fees from the Bill & Melinda Gates Foundation during the conduct of the study; personal fees from Anansi Health, personal fees from Avenir Health and personal fees from the Global Fund outside the submitted work. GG reports employment with the Bill & Melinda Gates Foundation. JE reports personal fees from the Bill & Melinda Gates Foundation during the conduct of the study; personal fees from the Bill & Melinda Gates Foundation outside the submitted work. TBH received grants and personal fees from the Bill & Melinda Gates Foundation during the conduct of the study; grants and personal fees from World Bank; grants from UNAIDS and the Rush Foundation; personal fees from the University of Washington, New York University and the Global Fund outside of the submitted work.

## Authors’ Contributions

GG, TBH and SJA conceived the study and designed the modelling analysis. JE reviewed the literature to inform the design of the study. SJA conducted the analyses and wrote the first draft of the manuscript. All authors reviewed and approved the final version.

## Supporting information


**Data S1.** Model description.
**Table S1.** The range of PAF values by sub‐population and HIV prevalence across locations of each epidemic type (groups 1 to 5).
**Table S2.** Proportion of sex acts using a condom by partnership type and modelled location.
**Table S3.** The modelled states describing the natural history of HIV infection and engagement with the treatment programme.
**Table S4.** List of parameter values used in specifying the model and their description
**Table S5.** ART programme‐related parameter values: Those parameters which are varied under the universe of futures are highlighted.
**Table S6.** Parameter bounds included in the model fitting.
**Figure S1.** Demonstration of Model Fit: A scatterplot of the modelled data versus the input data for a number of variables in each location.
