# Supplementary material for: The importance of local epidemic conditions in monitoring progress towards HIV epidemic control in Kenya: a modelling study
Source: J Int AIDS Soc. 2018 Nov 28;21(11):e25203. doi: 10.1002/jia2.25203 (PMC6260921; doi:10.1002/jia2.25203)
Supplement: Supplementary file 1 — Data S1. Model description. Table S1. The range of PAF values by sub‐population and HIV prevalence across locations of each epidemic type (groups 1 to 5). Table S2. Proportion of sex acts using a condom by partnership type and modelled location. Table S3. The modelled states describing the natural history of HIV infection and engagement with the treatment programme. Table S4. List of parameter values used in specifying the model and their description Table S5. ART programme‐related parameter values: Those parameters which are varied under the universe of futures are highlighted. Table S6. Parameter bounds included in the model fitting. Figure S1. Demonstration of Model Fit: A scatterplot of the modelled data versus the input data for a number of variables in each location. [file JIA2-21-e25203-s001.docx]

**Supplementary Materials**

Model Description

The model is described by a set of ordinary differential equations solved numerically using Euler’s Method. The counties and three major cities of Kenya (Nairobi, Mombasa, and Kisumu) are modelled independently (denoted as location l). Each county or city specific model is tailored to reflect the estimated mapped HIV prevalence and key indicators including circumcision rates and reported sexual behaviour. The sexually active population (15-49) is stratified by both sex and partner preference (k=1 heterosexual females, k=2 heterosexual males, and k=3 MSM) and reported risk behaviour (denoted as r). The model also distinguishes between those who receive PrEP and circumcision status in men and those who receive PrEP in women (intervention status denoted i).

In the analysis presented, we look specifically at indicators for FSW and MSM, to whom tailored services may typically be targeted, and the remaining ‘general population’ of heterosexual men and women. In specifying heterosexual behaviour in the model, a more detailed description of risk is used, with three risk groups are used based on the reported sexual behaviour from the DHS 2008/2009 (1). The highest risk group (risk group 1) is defined as those that report engaging in commercial sex in the past 12 months, and in women corresponds to the FSW group in the model. The medium risk group (risk group 2) report non-marital or non-cohabiting partnerships in the past 12 months. The fraction of the population remaining is considered low risk (risk group 3). General population men here refer to heterosexual men of all three risk groups, and general population women refer to women in groups 2 and 3.

We describe the behaviour of MSM in different locations through dividing the MSM population into three risk groups. High risk MSM are divided into two groups based on whether they form homosexual partnerships exclusively (risk group 1) or can form both heterosexual and homosexual partnerships (risk group 2). Risk group 3 MSM are assumed to be at lower risk with all individuals assumed to be bisexual. In county level models (corresponding to 45 modelled locations) only MSM in risk group 3 are included, as such all MSM are assumed to be both low risk and bisexual. In city level models (Nairobi, Mombasa, Kisumu) all three risk groups are included due to greater data availability to inform these groups. Further details of the specification of risk behaviour in the model can be found elsewhere (2).As discussed in the analysis, we use the relative contribution of the four different population groups (MSM, FSW, general population men and general population women) to the epidemic to classify each modelled location into one of five different epidemic types. Epidemics were classified into groups using k-means clustering based on the PAF values across subpopulations and HIV prevalence in each location. The characteristics of the groups are presented in Figure 1; we can see that locations within each group are relatively homogenous in their characteristics. In addition to the presentation of the characteristics of the groups in Figure 1, Table S1 below gives the range of PAF values across the subpopulations and prevalence levels in across locations for each epidemic type (Group 1-5).

Table S1. The range of PAF values by subpopulation and HIV prevalence across locations of each epidemic type (Group 1-5).

|  | **Group 1** | | **Group 2** | | **Group 3** | | **Group 4** | | **Group 5** | |
| --- | --- | --- | --- | --- | --- | --- | --- | --- | --- | --- |
|  | Range | | Range | | Range | | Range | | Range | |
| PAF FSW | 0.023 | 0.26 | 0.014 | 0.05 | 0.616 | 0.999 | 0.005 | 0.03 | 0.002 | 0.309 |
| PAF Low Risk Women | 0.745 | 0.831 | 0.438 | 0.63 | 0.008 | 0.531 | 0.551 | 0.56 | 0.861 | 0.997 |
| PAF Heterosexual Men | 0.695 | 0.825 | 0.438 | 0.63 | 0.669 | 0.999 | 0.565 | 0.67 | 0.873 | 0.999 |
| PAF MSM | 0.222 | 0.446 | 0.378 | 0.58 | 0.011 | 0.369 | 0.009 | 0.03 | 0.006 | 0.187 |
| HIV prevalence | 0.002 | 0.405 | 0.004 | 0.03 | 0.001 | 0.022 | 0.103 | 0.2 | 0 | 0.142 |

**Adaptation of Published Model** A number of extensions to our pre-existing model (full details available elsewhere (2)) were made to allow for a wider range of possible future outcomes and indicators to monitor. These include the explicit incorporation of condom use in the model and a more detailed representation of the treatment programme. These changes, and the refitting of the updated model, are described below.

Incorporating Condom Use into the Model

The model was altered such that transmission was modelled per sex act rather than per partnership, so that the effect of changing condom use could be determined. The probability of transmission in a partnership (β) is dependent on the number of sex acts in a partnership (n), the proportion of sex acts in which condoms were used (c) and the probability of transmission per sex act (α):

$$\beta_{r,r'}^{s^{'},i}=1-{(1-\alpha_{r,r^{'}}^{s^{'},i})}^{n_{r,r'}(1-c_{r,r'})}$$

with r the risk group of interest, r’ the risk group of the partner, s’ the infection state of the partner, and i the intervention status in the group of interest. The number of sex acts and proportion of sex acts in which condoms are used is dependent on whether it is a commercial, casual, long term heterosexual or homosexual partnership. As described before (2), the type of partnership (i.e. commercial, casual or long term heterosexual or homosexual) was classified based on the risk groups mixing (denoted by risk group r and the risk group of their partner r’) when forming that partnership. The parameters describing the number of sex acts by partnership type were included in the model fitting. The proportion of sex acts in which condoms were used is both location and partnership type specific. Data on condom use by location was taken from the Kenya County Profiles (3). Data on condom use by partnership type was taken from the 2014 Kenya AIDS Response Progress Report with estimates specific to homosexual, heterosexual commercial and casual partnerships (4). Low risk partnerships were assumed to have the same patterns of condom use as medium risk partnerships. To generate the location and partnership specific estimates of condom use, reported condom use by partnership type was scaled to reflect the location specific condom use estimates relative to the average condom use reported across locations. Where the scaled values exceeded 95% of sex acts protected by a condom it was capped at 95%.

Table S2. Proportion of sex acts using a condom by partnership type and modelled location.

| Location | Homosexual | Commercial | Low Risk | Location | Homosexual | Commercial | Low Risk |
| --- | --- | --- | --- | --- | --- | --- | --- |
| Baringo | 0.73 | 0.92 | 0.34 | **Marsabit** | 0.69 | 0.86 | 0.32 |
| Bomet | 0.86 | 0.95 | 0.39 | **Meru** | 0.63 | 0.78 | 0.29 |
| Bungoma | 0.61 | 0.76 | 0.28 | **Migori** | 0.81 | 0.95 | 0.37 |
| Busia | 0.73 | 0.92 | 0.34 | **Murang'a** | 0.57 | 0.71 | 0.26 |
| Embu | 0.87 | 0.95 | 0.40 | **Nakuru** | 0.84 | 0.95 | 0.39 |
| Garissa | 0.69 | 0.86 | 0.32 | **Nandi** | 0.66 | 0.82 | 0.30 |
| Homa Bay | 0.78 | 0.95 | 0.36 | **Narok** | 0.44 | 0.55 | 0.20 |
| Isiolo | 0.69 | 0.86 | 0.32 | **Nyamira** | 0.67 | 0.84 | 0.31 |
| Kajiado | 0.44 | 0.55 | 0.20 | **Nyandarua** | 0.50 | 0.63 | 0.23 |
| Kakamega | 0.72 | 0.90 | 0.33 | **Nyeri** | 0.86 | 0.95 | 0.39 |
| Elgeyo-Marakwet | 0.95 | 0.95 | 0.44 | **Samburu** | 0.54 | 0.67 | 0.25 |
| Kericho | 0.69 | 0.86 | 0.32 | **Siaya** | 0.69 | 0.86 | 0.32 |
| Kiambu | 0.72 | 0.90 | 0.33 | **Taita Taveta** | 0.83 | 0.95 | 0.38 |
| Kilifi | 0.76 | 0.95 | 0.35 | **Tana River** | 0.69 | 0.86 | 0.32 |
| Kirinyaga | 0.44 | 0.55 | 0.20 | **Tharaka** | 0.81 | 0.95 | 0.37 |
| Kisii | 0.66 | 0.82 | 0.30 | **Trans Nzoia** | 0.63 | 0.78 | 0.29 |
| Kisumu | 0.86 | 0.95 | 0.39 | **Turkana** | 0.69 | 0.86 | 0.32 |
| Kitui | 0.69 | 0.86 | 0.32 | **Uasin Gishu** | 0.80 | 0.95 | 0.37 |
| Kwale | 0.46 | 0.57 | 0.21 | **Vihiga** | 0.73 | 0.92 | 0.34 |
| Laikipia | 0.75 | 0.94 | 0.34 | **Wajir** | 0.69 | 0.86 | 0.32 |
| Lamu | 0.69 | 0.86 | 0.32 | **West Pokot** | 0.28 | 0.34 | 0.13 |
| Machakos | 0.58 | 0.73 | 0.27 | **Nairobi (City)** | 0.84 | 0.95 | 0.39 |
| Makueni | 0.55 | 0.69 | 0.25 | **Mombasa (City)** | 0.69 | 0.86 | 0.32 |
| Mandera | 0.69 | 0.86 | 0.32 | **Kisumu (City)** | 0.86 | 0.95 | 0.39 |

The probability of transmission per sex act was assumed to be 0.002 for heterosexual sex (men to women), 0.001 for heterosexual sex (women to men) and 0.0138 for homosexual sex (MSM) from those chronically infected with CD4>350 (5). This probability of transmission is modified depending on the infection state of the positive partner (with multipliers defined relative to the CD4>350 state baseline) and the intervention status of both partners. Those who are positive and are on treatment are assumed to be 85% less likely to transmit their infection onwards (6). This is incorporated into the probability of transmission by infection state. Those who are negative and are on PrEP, or negative men who are circumcised, are 75% and 60% respectively less likely to acquire infection (7, 8). This is also incorporated into the probability of transmission per sex act (α).

The Representation of the Treatment Programme

The model was revised to provide a more detailed representation of the treatment programme to allow for investigation of additional indicators, including those describing retention and viral suppression in the treated population.

Positive individuals may receive treatment via the ‘early’ or ‘late’ ART route. Early ART describes a route in which positive individuals are actively sought for enrolment in care, and applies to all those chronically infected, whilst late ART describes those of low CD4 counts (CD4<200) who present to services due to ill health.

Those who receive early ART are divided based on good or bad adherence (specified by the parameters $A_{h}$ and $A_{l}$ which describe the proportion with high adherence for those coming from high and low CD4 states respectively). Those who are good adherers are assumed to be virally suppressed. Those from high CD4 counts who are good adherers enter an initial ‘grace period’ whereby no one drops out of the treatment programme and there is no additional HIV mortality. This is to reflect the fact that such a route will positively select for those who are likely to remain engaged with the treatment programme. Following this grace period a fraction of individuals are at risk of disengaging from the treatment programme. Those on early ART who are ‘bad adherers’ are assumed to experience the same outcomes as those who join the late ART program. Those that receive ART via the early ART route but at low CD4 counts (CD4<200) are assumed to have the same outcomes of those receiving ART via the late ART route. Those who receive late ART are also divided based on good or bad adherence, and whether individuals can or cannot drop out from the treatment programme (with the proportion who can drop out defined by the parameter D, and the dropout rate by the parameter δ). Those that drop out may re-enter the CD4 200-350 category (with the proportion who can reinitiate defined by the parameter R), and so may re-enter the treatment programme via the early or late ART route. Bad adherers are assumed to have half the net survival time as good adherers.

Model Equations

The sexually active population (15-49, denoted a=1) is stratified by both sex and partner preference (k=1 heterosexual females, k=2 heterosexual males, and k=3 MSM) and reported risk behaviour (denoted as r). The model also distinguishes between those who receive PrEP and circumcision status in men and those who receive PrEP in women (intervention status denoted i). ART use and death are also described in the population aged 50+ (a= 2) however no further transmission is considered in this group. The natural history of HIV infection and treatment status are described by 21 states (s) (Table S3). The state variables are of the form $X_{r,i,a}^{s,l,k}.$ The ordinary differential equations below describe the movement between these states in the population aged 15-49 (a=1).

Table S3. The modelled states describing the natural history of HIV infection and engagement with the treatment programme. In state 1 individuals are HIV negative, states 2-7 reflect the natural history of HIV infection with individuals moving from acute phase through successive CD4 categories and states 8-21 reflect the different levels of engagement individuals may have with the treatment programme, based on their adherence to treatment, time on treatment and propensity to drop out of the programme.

| State | Description |
| --- | --- |
| $\boldsymbol{X}^{\boldsymbol{1}}$ | HIV negative |
| $\boldsymbol{X}^{\boldsymbol{2}}$ | HIV positive acute stage |
| $\boldsymbol{X}^{\boldsymbol{3}}$ | HIV positive with CD4>500 |
| $\boldsymbol{X}^{\boldsymbol{4}}$ | HIV positive with CD4 350-500 |
| $\boldsymbol{X}^{\boldsymbol{5}}$ | HIV positive with CD4 200-350 |
| $\boldsymbol{X}^{\boldsymbol{6}}$ | HIV positive with CD4 <200 |
| $\boldsymbol{X}^{\boldsymbol{7}}$ | HIV positive with CD4<200 prior to ART initiation |
| $\boldsymbol{X}^{\boldsymbol{8}}$ | Grace Period for Early ART from high CD4, good adherence, year 1 |
| $\boldsymbol{X}^{\boldsymbol{9}}$ | Good adherence, no drop out, year 1 |
| $\boldsymbol{X}^{\boldsymbol{10}}$ | Bad adherence, no drop out, year 1 |
| $\boldsymbol{X}^{\boldsymbol{11}}$ | Good adherence, no drop out, >1 year |
| $\boldsymbol{X}^{\boldsymbol{12}}$ | Bad adherence, no drop out, >1 year |
| $\boldsymbol{X}^{\boldsymbol{13}}$ | Good adherence, can drop out, year 1 |
| $\boldsymbol{X}^{\boldsymbol{14}}$ | Bad adherence, can drop out, year 1 |
| $\boldsymbol{X}^{\boldsymbol{15}}$ | Good adherence, can drop out, >1year |
| $\boldsymbol{X}^{\boldsymbol{16}}$ | Bad adherence, can drop out, >1year |
| $\boldsymbol{X}^{\boldsymbol{17}}$ | Dropped Out |
| $\boldsymbol{X}^{\boldsymbol{18}}$ | Grace Period for Early ART from high CD4 , no drop out, >1 year |
| $\boldsymbol{X}^{\boldsymbol{19}}$ | Grace Period for Early ART from high CD4 , can drop out, >1 year |
| $\boldsymbol{X}^{\boldsymbol{20}}$ | After Grace Period for Early ART, can drop out |
| $\boldsymbol{X}^{\boldsymbol{21}}$ | After Grace Period for Early ART, no drop out |

Table S4. List of parameter values used in specifying the model and their description

| $E_{r,i}^{l,k}$ | The number of individuals entering the population group (r,i,k) in location l |
| --- | --- |
| 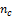 | The rate of circumcision in susceptible men |
| 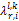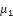 | The force of infection |
|  | The rate of aging from the 15-49 group |
| 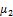 | The background rate of mortality in the population aged 50+ |
| 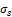 | The rate of progression between states |
| 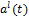 | The ART coverage for those receiving late ART |
| 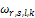 | The rate of early ART |
| 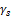 | The proportion of individuals who enter each CD4 state following acute stage |
| 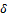 | The rate of drop out from the ART programme |
| 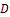 | the proportion of the population who can drop out of the ART programme |
| R | The proportion who drop out who can reinitiate |
| 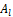 | The proportion of those from low CD4 states who are good adherers |
| 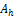 | The proportion of those from high CD4 who are good adherers. |
| 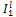 | The total number of individuals in the 15-49 population |
| 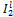 | The total number of individuals in the 50+ population |
| 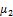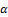 | The annual natural growth rate of the population |
| 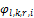 | The fraction of individuals entering the model in location l of the sex and partnership preference group k, risk group r and intervention group i |

**In susceptible (HIV negative) individuals:**

**In women (k=1):**

$\frac{{dX}_{r,i,1}^{1,l,1}}{dt}= E_{r,i}^{l,1}-\left( \lambda_{r,i}^{l,1}+\mu_{1} \right)X_{r,i,1}^{1,l,1}$

**In men (k=2, k=3):**

$\frac{{dX}_{r,1,1}^{1,l,k}}{dt}= E_{r,1}^{l,k}-(\lambda_{r,1}^{l,k}+\mu_{1})X_{r,1,1}^{1,l,k}{-n}_{c}X_{r,1,1}^{1,l,k}$

$\frac{{dX}_{r,2,1}^{1,l,k}}{dt}= E_{r,2}^{l,k}-\left( \lambda_{r,2}^{l,k}+\mu_{1} \right)X_{r,2,1}^{1,l,k}{+n}_{c}X_{r,1,1}^{1,l,k}$

$\frac{{dX}_{r,3,1}^{1,l,k}}{dt}= E_{r,3}^{l,k}-\left( \lambda_{r,3}^{l,k}+\mu_{1} \right)X_{r,3,1}^{1,l,k}{-n}_{c}X_{r,3,1}^{1,l,k}$

$\frac{{dX}_{r,4,1}^{1,l,k}}{dt}= E_{r,4}^{l,k}-(\lambda_{r,4}^{l,k}+\mu_{1})X_{r,4,1}^{1,l,k}{+n}_{c}X_{r,3,1}^{1,l,k}$

**In those HIV positive:**

$\frac{{dX}_{r,i,1}^{2,l,k}}{dt}={\lambda_{r,i}^{l,k}X}_{r,i,1}^{1,l,k}-(\mu_{1}+\sigma_{2})X_{r,i,1}^{2,l,k}$

$\frac{{dX}_{r,i,1}^{3,l,k}}{dt}=\gamma_{3}{\sigma_{2}X}_{r,i,1}^{2,l,k}-{\left( \mu_{1}+\sigma_{3}+\omega_{r,3,l,k} \right)X}_{r,i,1}^{3,l,k}$

$\frac{{dX}_{r,i,1}^{4,l,k}}{dt}={{\gamma_{4}{\sigma_{2}X}_{r,i,1}^{2,l,k}+\sigma}_{3}X}_{r,i,1}^{3,l,k}-(\mu_{1}+\sigma_{4}+\omega_{r,4,l,k})X_{r,i,1}^{4,l,k}$

$\frac{{dX}_{r,i,1}^{5,l,k}}{dt}={{\gamma_{5}{\sigma_{2}X}_{r,i,1}^{2,l,k}+{\delta R(X}_{r,i,1}^{13,l,k}+X_{r,i,1}^{14,l,k}+X_{r,i,1}^{15,l,k}+X_{r,i,1}^{16,l,k}+X_{r,i,1}^{20,l,k})+\sigma}_{4}X}_{r,i,1}^{4,l,k}-{(\mu_{1}+\sigma_{5}+\omega_{r,5,l,k})X}_{r,i,1}^{5,l,k}$

$\frac{{dX}_{r,i,1}^{6,l,k}}{dt}=(1-a^{l}(t))({{\gamma_{6}{\sigma_{2}X}_{r,i,1}^{2,l,k}+\sigma}_{5}X}_{r,i,1}^{5,l,k})-{(\mu_{1}+\sigma_{6}+\omega_{r,6,l,k})X}_{r,i,1}^{6,l,k}$

$\frac{{dX}_{r,i,1}^{7,l,k}}{dt}=a^{l}(t)({{\gamma_{6}{\sigma_{2}X}_{r,i,1}^{2,l,k}+\sigma}_{5}X}_{r,i,1}^{5,l,k})-{(\mu_{1}+\sigma_{6}+\sigma_{7}+\omega_{r,7,l,k})X}_{r,i,1}^{7,l,k}$

$\frac{{dX}_{r,i,1}^{8,l,k}}{dt}=A_{h}({\omega_{r,3,l,k}X}_{r,i,1}^{3,l,k}+\omega_{r,4,l,k}X_{r,i,1}^{4,l,k}+{\omega_{r,5,l,k}X}_{r,i,1}^{5,l,k})-{(\mu_{1}+\sigma_{8})X}_{r,i,1}^{8l,k}$

$\frac{{dX}_{r,i,1}^{9,l,k}}{dt}={(1-D)A}_{l}{(\sigma_{7}X}_{r,i,1}^{7,l,k}+ ({\omega_{r,6,l,k}X}_{r,i,1}^{6,l,k}+{\omega_{r,7,l,k}X}_{r,i,1}^{7,l,k}){)-(\mu_{1}+p_{9}+P)X}_{r,i,1}^{9,l,k}$

$\frac{{dX}_{r,i,1}^{10,l,k}}{dt}$=${\left( 1-D \right)(1-A}_{l}){(\sigma_{7}X}_{r,i,1}^{7,l,k}+ \left( {\omega_{r,6,l,k}X}_{r,i,1}^{6,l,k}+{\omega_{r,7,l,k}X}_{r,i,1}^{7,l,k} \right))+{(1-D)(1-A}_{h})({\omega_{r,3,l,k}X}_{r,i,1}^{3,l,k}+\omega_{r,4,l,k}X_{r,i,1}^{4,l,k}+{\omega_{r,5,l,k}X}_{r,i,1}^{5,l,k})-{(\mu_{1}+p_{10}+P)X}_{r,i,1}^{10,l,k}$

$\frac{{dX}_{r,i,1}^{11,l,k}}{dt}={PX}_{r,i,1}^{9,l,k}$ $-{(\mu_{1}+p_{11})X}_{r,i,1}^{11,l,k}$

$\frac{{dX}_{r,i,1}^{12,l,k}}{dt}={PX}_{r,i,1}^{10,l,k}$ $-{(\mu_{1}+p_{12})X}_{r,i,1}^{12,l,k}$

$\frac{{dX}_{r,i,1}^{13,l,k}}{dt}=$ ${DA}_{l}{(\sigma_{7}X}_{r,i,1}^{7,l,k}+ \left( {\omega_{r,6,l,k}X}_{r,i,1}^{6,l,k}+{\omega_{r,7,l,k}X}_{r,i,1}^{7,l,k} \right))-{(\mu_{1}+p_{13}+\delta+P)X}_{r,i,1}^{13,l,k}$

$$\frac{{dX}_{r,i,1}^{14,l,k}}{dt}={D(1-A}_{l}){(\sigma_{7}X}_{r,i,1}^{7,l,k}+ \left( {\omega_{r,6,l,k}X}_{r,i,1}^{6,l,k}+{\omega_{r,7,l,k}X}_{r,i,1}^{7,l,k} \right))+{D(1-A}_{h})({\omega_{r,3,l,k}X}_{r,i,1}^{3,l,k}+\omega_{r,4,l,k}X_{r,i,1}^{4,l,k}+{\omega_{r,5,l,k}X}_{r,i,1}^{5,l,k})-{(\mu_{1}+p_{14}+\delta+P)X}_{r,i,1}^{14,l,k}$$

$\frac{{dX}_{r,i,1}^{15,l,k}}{dt}=$ ${PX}_{r,i,1}^{13,l,k}-{(\mu_{1}+p_{15}+\delta)X}_{r,i,1}^{15,l,k}$

$\frac{{dX}_{r,i,1}^{16,l,k}}{dt}=$ ${PX}_{r,i,1}^{14,l,k}-{(\mu_{1}+p_{16}+\delta)X}_{r,i,1}^{16,l,k}$

$\frac{{dX}_{r,i,1}^{17,l,k}}{dt}=$ $(1-R)\delta(X_{r,i,1}^{13,l,k}+X_{r,i,1}^{14,l,k}+X_{r,i,1}^{15,l,k}+X_{r,i,1}^{16,l,k}+X_{r,i,1}^{20,l,k})-{(\mu_{1}+p_{17})X}_{r,i,1}^{17,l,k}$

$\frac{{dX}_{r,i,1}^{18,l,k}}{dt}=$ $(1-D){\sigma_{8}X}_{r,i,1}^{8l,k}-{(\mu_{1}+\sigma_{18})X}_{r,i,1}^{18,l,k}$

$\frac{{dX}_{r,i,1}^{19,l,k}}{dt}=D{\sigma_{8}X}_{r,i,1}^{8l,k}-{(\mu_{1}+\sigma_{19})X}_{r,i,1}^{19,l,k}$

$\frac{{dX}_{r,i,1}^{20,l,k}}{dt}=$ ${\sigma_{19}X}_{r,i,1}^{19,l,k}-{(\mu_{1}+p_{20}+\delta)X}_{r,i,1}^{20,l,k}$

$\frac{{dX}_{r,i,1}^{21,l,k}}{dt}=$ ${\sigma_{18}X}_{r,i,1}^{18,l,k}-{(\mu_{1}+p_{21})X}_{r,i,1}^{21,l,k}$

Where $E_{r,i}^{l,k}$ is the number of individuals entering the population group (r,i,k) in location l , $n_{c}$ the rate of circumcision in susceptible men, $\lambda_{r,i}^{l,k}$ the force of infection, $\mu_{1}$ the rate of aging from the 15-49 group, $\sigma_{s}$ the rate of progression between states, $a^{l}(t)$ the ART coverage for those receiving late ART and $\omega_{r,s,l,k}$ the rate of early ART. Following the acute stage of infection, not all individuals pass through all CD4 states sequentially, with some entering later stages with lower CD4 counts immediately. Here the proportion of individuals who enter each state following acute stage is defined by the parameter $\gamma_{s}.$

Where $\delta$is the rate of drop out from the ART programme, $D$ is the proportion of the population who can drop out of the ART programme, R is the proportion who drop out who can reinitiate, $A_{l}$is the proportion of those from low CD4 states who are good adherers, and $A_{h}$ is the proportion of those from high CD4 who are good adherers.

The number of individuals entering each subpopulation $E_{r,i}^{l,k}$is calculated from the total number of individuals who enter the model at age 15 in each location l, denoted ($K_{l}),$ given by :

$$I_{1}^{l}=\sum_{r} \sum_{i} \sum_{k} \sum_{s} X_{r,i}^{s,l,k}$$

$$I_{2}^{l}=\sum_{s} Y_{l}^{s}$$

$$K_{l}=\mu_{2}I_{2}^{l}+\alpha(I_{1}^{l}+I_{2}^{l})$$

Where$I_{1}^{l}$is the total number of individuals in the 15-49 population,$I_{2}^{l}$ is the total number of individuals in the 50+ population, $\mu_{2}$ is the death rate in the population aged 50+ and $\alpha$ the annual natural growth rate of the population assumed to be 3% based on data from the World Bank (9). The total number of individuals entering the population$K_{l}$is divided between the relevant subgroups according to the assumed distribution of sex and partner preference (k), risk behaviour (r) and intervention status (i) in the population in location (l). All individuals who enter the model are assumed to be susceptible, that is we do not consider infections that were acquired prior to the age of 15. The number of individuals entering each subpopulation$E_{r,i}^{l,k}$is given by:

$$E_{r,i}^{l,k}=K_{l}\varphi_{l,k,r,i}$$

where $\varphi_{l,k,r,i}$ is the fraction of individuals entering the model in location l of the sex and partnership preference group k, risk group r and intervention group i. It is assumed that half of the population entering the model are female and half male, and that 3% of men are MSM.

In the population aged 50+ (a=2) the equations are as below. As no transmission is considered in this age group, intervention, risk and partner preference groups from the younger age group are amalgamated, with the state variable of the form$Y_{l}^{s}$ .

$\frac{{dY}_{l}^{1}}{dt}=\sum_{r} \sum_{i} \sum_{k} X_{r,i,1}^{1,l,k}\mu_{1}-\mu_{2}Y_{l}^{1}$

$\frac{{dY}_{l}^{2}}{dt}=\sum_{r} \sum_{i} \sum_{k} X_{r,i,1}^{2,l,k}\mu_{1}-(\mu_{2}+\sigma_{2})Y_{l}^{2}$

$\frac{{dY}_{l}^{3}}{dt}=\sum_{r} \sum_{i} \sum_{k} X_{r,i,1}^{3,l,k}\mu_{1}+{\sigma_{2}Y}_{l}^{2}-{{(\mu}_{2}+\sigma_{3})Y}_{l}^{3}$

$\frac{{dY}_{l}^{4}}{dt}=\sum_{r} \sum_{i} \sum_{k} X_{r,i,1}^{4,l,k}\mu_{1}+\sigma_{3}Y_{l}^{3}-{(\mu}_{2}+\sigma_{4})Y_{l}^{4}$

$\frac{{dY}_{l}^{5}}{dt}=\sum_{r} \sum_{i} \sum_{k} X_{r,i,1}^{5,l,k}{\mu_{1}+\sigma}_{4}Y_{l}^{4}-(\mu_{2}{+\sigma_{5})Y}_{l}^{5}$

$\frac{{dY}_{l}^{6}}{dt}=\sum_{r} \sum_{i} \sum_{k} X_{r,i,1}^{6,l,k}{\mu_{1}+(1-a^{l}(t))\sigma}_{5}Y_{l}^{5}-{\left( \mu_{2}+\sigma_{6} \right)Y}_{l}^{6}$

$\frac{{dY}_{l}^{7}}{dt}=\sum_{r} \sum_{i} \sum_{k} X_{r,i,1}^{7,l,k}{\mu_{1}+a^{l}(t)\sigma}_{5}Y_{l}^{5}-(\mu_{2}+\sigma_{6}+\sigma_{7})Y_{l}^{7}$

$\frac{{dY}_{l}^{8}}{dt}=\sum_{r} \sum_{i} \sum_{k} X_{r,i,1}^{8,l,k}{\mu_{1}+\sigma}_{7}Y_{l}^{7}-{(\mu}_{2}+\sigma_{8})Y_{l}^{8}$

$\frac{{dY}_{l}^{9}}{dt}=\sum_{r} \sum_{i} \sum_{k} X_{r,i,1}^{9,l,k}\mu_{1}{+\left( 1-D \right)A}_{l}{\sigma_{7}Y}_{l}^{7}-{(\mu_{2}+p_{9}+P)Y}_{l}^{9}$

$\frac{{dY}_{l}^{10}}{dt}=\sum_{r} \sum_{i} \sum_{k} X_{r,i,1}^{10,l,k}\mu_{1}{+\left( 1-D \right)(1-A}_{l}){\sigma_{7}Y}_{l}^{7}-{(\mu_{2}+p_{10}+P)Y}_{l}^{10}$

$\frac{{dY}_{l}^{11}}{dt}=\sum_{r} \sum_{i} \sum_{k} X_{r,i,1}^{11,l,k}\mu_{1}{-(\mu_{2}+p_{11})Y}_{l}^{11}$

$\frac{{dY}_{l}^{12}}{dt}=\sum_{r} \sum_{i} \sum_{k} X_{r,i,1}^{12,l,k}\mu_{1}{-(\mu_{2}+p_{12})Y}_{l}^{12}$

$\frac{{dY}_{l}^{13}}{dt}=\sum_{r} \sum_{i} \sum_{k} X_{r,i,1}^{13,l,k}\mu_{1}{+DA}_{l}{\sigma_{7}Y}_{l}^{7}-{(\mu_{2}+p_{13}+P+\delta)Y}_{l}^{13}$

$\frac{{dY}_{l}^{14}}{dt}=\sum_{r} \sum_{i} \sum_{k} X_{r,i,1}^{14,l,k}\mu_{1}{+D(1-A}_{l}){\sigma_{7}Y}_{l}^{7}-{(\mu_{2}+p_{14}+P+\delta)Y}_{l}^{14}$

$\frac{{dY}_{l}^{15}}{dt}=\sum_{r} \sum_{i} \sum_{k} X_{r,i,1}^{15,l,k}\mu_{1}{+{PY}_{l}^{13}-(\mu_{2}+p_{15}+\delta)X}_{l}^{15}$

$\frac{{dY}_{l}^{16}}{dt}=\sum_{r} \sum_{i} \sum_{k} X_{r,i,1}^{16,l,k}\mu_{1}{+{PY}_{l}^{14}-(\mu_{2}+p_{16}+\delta)Y}_{l}^{16}$

$\frac{{dY}_{l}^{17}}{dt}=\sum_{r} \sum_{i} \sum_{k} X_{r,i,1}^{17,l,k}\mu_{1}$+$\delta(Y_{l}^{13}+Y_{l}^{14}+Y_{l}^{15}+Y_{l}^{16}+Y_{l}^{20})-(\mu_{2}+p_{17})Y_{l}^{17}$

$\frac{{dY}_{l}^{18}}{dt}=\sum_{r} \sum_{i} \sum_{k} X_{r,i,1}^{18,l,k}\mu_{1}$ + ${{(1-D)\sigma}_{8}Y}_{l}^{8} -{(\mu_{2}+\sigma_{18})Y}_{l}^{18}$

$\frac{{dY}_{l}^{19}}{dt}=\sum_{r} \sum_{i} \sum_{k} X_{r,i,1}^{19,l,k}\mu_{1}+D\sigma_{8}Y_{l}^{8}-(\mu_{2}+\sigma_{19})Y_{l}^{19}$

$\frac{{dY}_{l}^{20}}{dt}=\sum_{r} \sum_{i} \sum_{k} X_{r,i,1}^{20,l,k}\mu_{1}$ $+\sigma_{19}Y_{l}^{19}-(\mu_{2}+p_{20}+\delta)Y_{l}^{20}$

$\frac{{dX}_{l}^{21}}{dt}=\sum_{r} \sum_{i} \sum_{k} X_{r,i,1}^{21,l,k}\mu_{1}+$ ${\sigma_{18}Y}_{l}^{18}-{(\mu_{1}+p_{21})Y}_{l}^{21}$

Where $\sigma_{s}$ is the rate of progression between states, $a^{l}(t)$ the coverage of ART in location l,$\mu_{1}$ the rate of aging from the 15-49 population into the 50+ population, and $\mu_{2}$ the background rate of mortality in the population aged 50+. Prevention interventions are only available to the sexually active (15-49) population and as such early ART and circumcision are not considered in the 50+ population. We assume no reinitiation following drop out from treatment in those who are 50+.

Table S5. ART Programme Related Parameter Values: Those parameters which are varied under the universe of futures are highlighted.

| Parameter | Description | Value |
| --- | --- | --- |
| Rate of Death in Those on ART- Dependent on Good/Bad Adherence | | |
| $\boldsymbol{p}_{\boldsymbol{11}}$ | Good Adherence | 1/θ  NB Net Survival Time on ART (θ) is Varied Under the Universe of Futures |
| $\boldsymbol{p}_{\boldsymbol{12}}$ | Bad Adherence | 1/(0.5*θ) |
| $\boldsymbol{p}_{\boldsymbol{13}}$ | Good Adherence | 1/θ |
| $\boldsymbol{p}_{\boldsymbol{14}}$ | Bad Adherence | 1/(0.5*θ) |
| $\boldsymbol{p}_{\boldsymbol{15}}$ | Good Adherence | 1/θ |
| $\boldsymbol{p}_{\boldsymbol{16}}$ | Bad Adherence | 1/(0.5*θ) |
| $\boldsymbol{p}_{\boldsymbol{17}}$ | Dropped Out | 1/(5.84) |
| $\boldsymbol{p}_{\boldsymbol{20}}$ | Good Adherence | 1/θ |
| $\boldsymbol{p}_{\boldsymbol{21}}$ | Good Adherence | 1/θ |
| ART Programme Related Parameters | | |
| D | Proportion who can drop out | Varied Under the Universe of Futures |
| $\boldsymbol{A}_{\boldsymbol{l}}$ | Proportion with good adherence from low CD4 | Varied Under the Universe of Futures |
| $\boldsymbol{A}_{\boldsymbol{h}}$ | Proportion with good adherence from high CD4 | Varied Under the Universe of Futures |
| δ | Drop Out Rate | Varied Under the Universe of Futures |
| R | Proportion of those who can drop out who can reinitiate | Varied Under the Universe of Futures |
| P | Rate of progress from year 1 of treatment | 1 |
| $\boldsymbol{\sigma}_{\boldsymbol{6}}$ | Rate of death due to AIDS | 1/2.3 |
| $\boldsymbol{\sigma}_{\boldsymbol{7}}$ | Rate of starting ART | 1/0.5 |
| $\boldsymbol{\sigma}_{\boldsymbol{8}}$ | Rate of progress from First Year Early ART | 1 |
| $\boldsymbol{\sigma}_{\boldsymbol{18}}$ | Rate of progress from Grace Period for Early ART Good Adherers | 1/3.6 |
| $\boldsymbol{\sigma}_{\boldsymbol{19}}$ | Rate of progress from Grace Period for Early ART Good Adherers | 1/3.6 |

Refitting the model

The updated model was refit for each county using the same approach as described previously (2). Briefly, the Nelder-Mead Optimisation algorithm was used to find the parameter set which maximises the likelihood of the model realisation with respect to available data. The model was fit to available data on prevalence, the level of male circumcision in the population, and treatment coverage. Latin hypercube sampling from within the boundaries defined was used to provide 25 seeds to avoid confinement in local optima. The parameters which were included in the fitting process, and their associated ranges are described in Table S6. The resulting model fits are presented in Figure S1.

Table S6. Parameter bounds included in the model fitting.

| Parameter | Lower Bound | Upper Bound |
| --- | --- | --- |
| Mean Partner Change Rate in Men | 1 | 3 |
| Mean Partner Change Rate in Women | 1 | 3 |
| Relative Risk Casual Sex Men | 2 | 20 |
| Relative Risk Very High Risk Sex Men | 30 | 300 |
| Relative Risk Casual Sex Women | 2 | 20 |
| Relative Risk Very High Risk Sex Women | 30 | 300 |
| Number of Sex Acts Low Risk Partnerships | 100 | 300 |
| Number of Sex Acts MSM | 10 | 100 |
| Number of Sex Acts Commercial | 1 | 10 |
| Number of Sex Acts Casual | 10 | 100 |
| Number of Sex Acts High Risk | 1 | 10 |

**Figure S1: Demonstration of Model Fit: A scatterplot of the modelled data versus the input data for a number of variables in each location.**

1. A scatterplot of the modelled HIV prevalence (y axis) versus the input data taken from the interpolated DHS 2008/2009 map (x axis) for each location (1). Dashed lines provide the 95% confidence intervals for the data, based on an assumed sample size of 300 people. The colour scheme used in this plot is to highlight the distribution of the data for ease of reading.
2. A scatterplot of the modelled HIV prevalence in women (y axis) versus the input data taken from the interpolated DHS 2008/2009 map (x axis) for each location (1). Dashed lines provide the 95% confidence intervals for the data, based on an assumed sample size of 150 people.
3. A scatterplot of the modelled HIV prevalence in men (y axis) versus the input data taken from the interpolated DHS 2008/2009 map (x axis) for each location (1). Dashed lines provide the 95% confidence intervals for the data, based on an assumed sample size of 150 people.
4. A scatterplot of the modelled ART coverage (y axis) against the input ART coverage data taken from official reports (x axis) for each location (3). Dashed lines provide the 95% confidence intervals for the data, based on an assumed sample size of 200 for measurement of ART coverage. The colour scheme used in this plot is to highlight the distribution of the data for ease of reading.
5. A scatterplot of the modelled percentage of men circumcised (y axis) versus the input data on male circumcision taken from official reports (x axis) for each location (3). Dashed lines provide the 95% confidence intervals for the data, based on an assumed sample size of 150 for measurement of the percentage of men circumcised. The colour scheme used in this plot is to highlight the distribution of the data for ease of reading.
6. A scatterplot of the modelled percentage of men in each risk group (y axis) versus the estimated percentage in each risk group as indicated by the input data (x axis) for each location. The province level estimate of the distribution of risk behaviours in men from the 2008/2009 DHS survey was used to inform the distribution of risk behaviours in the heterosexual population (1). The percentage of men who are MSM (3%) was fixed in the model and is based on a review of the literature (10). The green points correspond to the percentage of men who report being clients of sex workers, the blue points correspond to the percentage of men who report casual partners and red points correspond to the remaining percentage of the male population who are considered at low risk. The magenta point represents the proportion of men who are MSM.
7. A scatterplot of the modelled percentage of women in each risk group (y axis) versus the province level estimate of the distribution of risk behaviours in women from the 2008/2009 DHS survey (x axis) for each location (1). The green points correspond to the percentage of women who report commercial sex, the blue points correspond to the percentage of women who report casual partners and red points correspond to the remaining percentage of the female population who are considered at low risk.
8. A scatterplot of the modelled MSM: heterosexual men ratio (y axis) versus the input estimate of this ratio (x axis) for each location. The line of best fit demonstrates that we assume that MSM have approximately double the HIV prevalence of heterosexual men in all locations, this is based on a review of the literature (11).
9. A scatterplot of the modelled prevalence among high risk MSM (y axis) versus the observed prevalence in high risk MSM (x axis) for each location (12-14). Only three data points are present as high risk MSM are included in the city models (Nairobi, Mombasa and Kisumu City only). Dashed lines provide the estimated 95% confidence intervals for the data, based on an assumed sample size of 50 individuals.


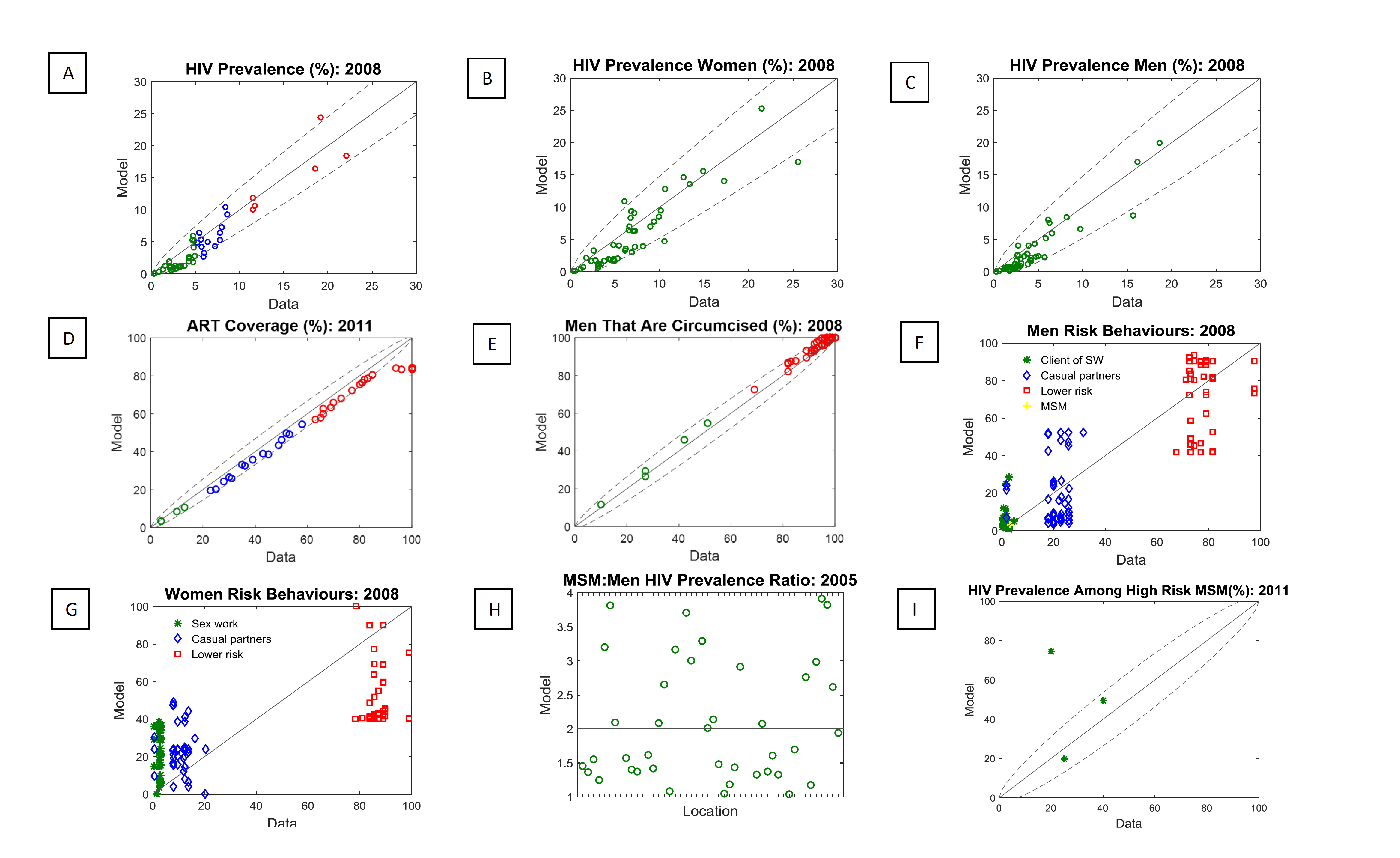


**References**

1. Kenya National Bureau of Statistics (KNBS) and ICF Macro. (2010) Kenya Demographic and Health Survey 2008-09. Calverton, Maryland. http://dhsprogram.com/pubs/pdf/fr229/fr229.pdf. accessed: 4 December 2015.

2. Anderson SJ, Cherutich P, Kilonzo N, Cremin I, Fecht D, Kimanga D, et al. Maximising the effect of combination HIV prevention through prioritisation of the people and places in greatest need: a modelling study. Lancet. 2014;384(9939):249-56.

3. National AIDS and STI Control Programme (NASCOP) (2013). Kenya, County HIV Service Delivery Profiles. http://www.faces-kenya.org/wp-content/uploads/2013/09/THE-FINAL-COUNTY-PROFILES-REPORT-FOR-KENYA-revised_2.pdf. accessed: 4 December 2015.

4. Kenya National AIDS Control Council (NACC). Kenya AIDS Response Progress Report 2014.

5. Cremin Í, Hallett TB. Estimating the range of potential epidemiological impact of pre-exposure prophylaxis: run-away success or run-away failure? AIDS. 2015;29(6).

6. Cohen MS, Chen YQ, McCauley M, Gamble T, Hosseinipour MC, Kumarasamy N, et al. Prevention of HIV-1 Infection with Early Antiretroviral Therapy. New England Journal of Medicine. 2011;365(6):493-505.

7. Cremin I, Alsallaq R, Dybul M, Piot P, Garnett G, Hallett TB. The new role of antiretrovirals in combination HIV prevention: a mathematical modelling analysis. AIDS (London, England). 2013;27(3):447-58.

8. Auvert B, Taljaard D, Lagarde E, Sobngwi-Tambekou J, Sitta R, Puren A. Randomized, controlled intervention trial of male circumcision for reduction of HIV infection risk: the ANRS 1265 Trial. PLoS Med. 2005;2(11):e298.

9. The World Bank. Population growth (annual %). http://data.worldbank.org/indicator/SP.POP.GROW. accessed: 4 December 2015.

10. Caceres CF, Konda K, Segura ER, Lyerla R. Epidemiology of male same-sex behaviour and associated sexual health indicators in low- and middle-income countries: 2003-2007 estimates. Sexually transmitted infections. 2008;84 Suppl 1:i49-i56.

11. Angala P, Parkinson A, Kilonzo N, Natecho A, M T, editors. Men who have sex with men (MSM) as presented in VCT data in Kenya. AIDS 2006-XVI International AIDS Conference; 2006; Toronto, ON, Canada.

12. Sanders EJ, Graham SM, Okuku HS, van der Elst EM, Muhaari A, Davies A, et al. HIV-1 infection in high risk men who have sex with men in Mombasa, Kenya. Aids. 2007;21(18):2513-20.

13. National AIDS and STI Control Programme (NASCOP) (2012). MARPs Surveillance Report 2012.

14. McKinnon LR, Gakii G, Juno JA, Izulla P, Munyao J, Ireri N, et al. High HIV risk in a cohort of male sex workers from Nairobi, Kenya. Sexually transmitted infections. 2014;90(3):237-42.
